# Time-limited cryomapping during tachycardia: improved long-term outcomes for cryoablation of AVNRT

**DOI:** 10.1007/s10840-016-0144-3

**Published:** 2016-05-25

**Authors:** Paula L. S. Eryazici, Mansour Razminia, Oliver D’Silva, Jaime R. Chavez, Ferah D. Ciftci, Marianne Turner, Theodore Wang, Terry A. Zheutlin, Richard F. Kehoe

**Affiliations:** Advocate Illinois Masonic Medical Center, 3000 N. Halsted St., Suite 803, Chicago, IL 60657 USA

**Keywords:** AVNRT, Cryoablation, Cryomapping, Time to effect, Time to tachycardia termination

## Abstract

**Purpose:**

Cryothermal ablation (CTA) for atrioventricular nodal reentrant tachycardia (AVNRT) is considered safer than radiofrequency ablation (RFA) since it eliminates the risk of inadvertent AV block. However, it has not been widely adopted due to high late recurrence rate (LRR). In an effort to improve LRR, we evaluated a new approach to cryothermal mapping (CTM): “time to tachycardia termination” (TTT).

**Methods:**

This single-center study had 88 consecutive patients who underwent CTA using TTT for AVNRT. The CTA catheter was positioned in sinus rhythm at the posteroseptal tricuspid annulus, and then AVNRT was induced. The CTA target site was identified by prompt tachycardia termination in ≤20 s during CTM. Procedural success was defined as no inducible AVNRT and ≤1 single AV nodal echoes.

**Results:**

Acute procedural success was achieved in 87 of 88 patients (98.9 %) and was similar to prior studies for both CTA and RFA. No permanent AV block was observed. LRR was 3.7 % at a mean follow-up of 19.7 months. LRR was equivalent to that commonly reported for RFA and improved when compared to conventional CTA.

**Conclusion:**

TTT for CTA of AVNRT provides enhanced safety and similar long-term efficacy when compared to RFA. Based upon this experience, TTT provides an enhancement to conventional CTA that appears to result in improved long-term outcomes. In light of these findings, it seems reasonable to undertake additional randomized trials to determine whether RFA or CTA using TTT is the optimal approach for the catheter ablation of AVNRT.

## Introduction

Radiofrequency (RF) catheter ablation is generally considered the preferred treatment option for patients with symptomatic atrioventricular nodal reentrant tachycardia (AVNRT) [1–3]. While its use is associated with excellent short and long-term success rates, a major drawback is the risk of inadvertent permanent AV block. This complication has been reported in up to 2.0 % of patients [3–6] and leaves them pacemaker-dependent for life. Given that patients with AVNRT are often young [1] and free of structural heart disease, this can be a life-altering complication.

Cryothermal ablation (CTA) is an alternative to radiofrequency ablation (RFA) with a similar acute procedural efficacy averaging 95 % that also offers the advantage of being virtually devoid of the risk of permanent AV block. The initial experience with CTA for AVNRT was reported in the multicenter prospective FROSTY trial. None of the 103 patients with AVNRT that underwent CTA developed inadvertent AV block that required permanent pacemaker implantation [7]. Subsequent studies have confirmed this finding in over 1000 patients [8–13].

Despite this noteworthy safety advantage, CTA has not been broadly adopted as the ablative technique of choice since its use has been associated with a higher risk of late arrhythmia recurrence [10, 13, 14], averaging 9–10 % for CTA compared to only 3–4 % for RFA based on the most recent studies [2, 3, 13, 15].

The inherent safety of CTA derives primarily from the reversibility of the cryothermal lesion during the first 30–60 s of cooling when lesion temperature is limited to a nadir of −30 °C. During this phase of cooling, the targeted tissue becomes temporarily unable to conduct, providing the operator assurance that irreversible CTA at that site will be effective and unlikely to create permanent AV block. The process of CTA target site selection is broadly referred to as cryothermal mapping (CTM). A variety of CTM end-points have been employed to determine whether a site is suitable for permanent CTA. These include (1) interruption of slow pathway conduction during CTM; (2) inability to reinduce AVNRT in a previously inducible patient; and (3) termination of sustained AVNRT during CTM.

In all previously reported large studies, however, the exact method employed for CTM was uncontrolled and left to the discretion of the investigator. Similarly, the time allowed to achieve the desired CTM effect was uncontrolled, often lengthy, and usually not reported. In some studies, a target site could be deemed appropriate for permanent CTA even when the desired effect occurred as late as 80 s into CTM [13]. Thus, it is possible that the higher late recurrence rate (LRR) often reported in prior CTA studies could be related to the CTM methodology employed. As an example on how the details of the CTM technique can actually have a significant impact on outcomes, improved CTA results have been noted when target site selection was guided by time-limited CTM, a feature that has often been referred to as “time to effect” [12, 16, 17].

The objective of our study was to determine if a more rigorously applied time-limited criterion for CTM success, namely time to tachycardia termination (TTT), would provide improved LRR for adult patients undergoing CTA for AVNRT. Based on the work of previous investigations [12, 16, 17], we defined a successful TTT as the termination of AVNRT due to slow pathway block within 20 s of the onset of CTM.

Given the excellent safety profile of CTA, if the LRR seen with its use could be improved to match that of RFA, it is reasonable to propose that CTA should become the modality of choice for patients undergoing catheter ablation for AVNRT.

## Methods

### Study design and patient selection

This study enrolled patients with symptomatic AVNRT who underwent CTA at our center between February 2005 and June 2011. We attempted to evaluate the utility of TTT during CTA in 94 consecutive patients. Sustained AVNRT was successfully induced in 88 of these 94 patients (94 %). The utility of TTT as a guide to CTM could therefore be evaluated in these 88 patients, and they comprised our study population. In the 6 patients in whom sustained AVNRT could not be induced, conventional CTM techniques applied during atrial extrastimulus testing was performed. In all 6 patients, CTA was successful and none required transition to RFA.

This study was approved by our local institutional review board. The data collected included patient demographics, arrhythmia characteristics, electrophysiologic study results, procedural details, complications, as well as acute and long-term outcomes.

### Electrophysiologic study and cryoablation procedure

CTA was performed with a 7-French 6-mm electrode tip catheter (Freezor Xtra®, CryoCath Technologies, Montreal, QC, Canada). In all patients, the target for CTA was the AV nodal slow pathway, whether it was utilized in the antegrade or retrograde direction. Procedure duration was measured from the first venous sheath placement until final sheath removal.

All procedures were performed by one of three operators. If CTA was unsuccessful, ablative therapy was transitioned to the RFA approach.

Electrophysiologic study included the placement of multiple electrode catheters in the standard positions. After recording baseline intervals, antegrade and retrograde properties were assessed using standard atrial and ventricular pacing and extrastimulus techniques. Up to three atrial extrastimuli at multiple cycle lengths were employed. If sustained tachycardia (≥90 s duration) could not be induced, repeat stimulation was undertaken during isoproterenol infusion. The reproducibility of tachycardia induction was confirmed by requiring three separate inductions of ≥90 s duration. AVNRT as the tachycardia mechanism was confirmed by previously described standard techniques [18]. Since December of 2010, all ablations at our institution have been performed without fluoroscopic guidance [19]. Catheter positioning was guided by an electro-anatomic mapping (EAM) system (EnSite NavX^TM^, St. Jude Medical, St. Paul, MN, USA), Therefore, fluoroscopy times are not reported.

### Cryomapping and cryoablation techniques

After confirmation of the reproducible induction of sustained AVNRT, the cryoablation catheter was positioned at the posteromedial tricuspid annulus during sinus rhythm. A site yielding a distal electrode recording with an A:V ratio of approximately 1:3 and/or a local slow pathway electrogram was considered suitable for CTM.

Sustained AVNRT was again induced. Potential target sites for permanent CTA were identified by the ability to terminate AVNRT with slow pathway block within 20 s of CTM application. Once a suitable target was identified by tachycardia termination, CTA was continued for a total application time of 4 min targeting a peak negative temperature of −70 °C.

During CTA application, repeated attempts to induce AVNRT and to assess the integrity of fast pathway conduction were undertaken. If tachycardia remained non-inducible and fast pathway function remained intact, 3 additional “consolidating” lesions guided by EAM were applied immediately adjacent to the successful site, in an effort to minimize the extent of the posteroseptal area exposed to permanent injury.

During these “consolidating” applications, AV conduction was closely monitored and repeated attempts to assess inducibility were undertaken.

If sustained AVNRT could not be terminated within 20 s, a different site would be selected for CTM. Additionally, if AV conduction became impaired or re-induction of AVNRT occurred, an alternate site for CTM was identified. If tachycardia remained non-inducible after the last consolidation application, a 30-min waiting period was required. Thereafter, a repeat EP study before and after isoproterenol infusion was undertaken. Procedural end-points for success were defined as non-inducibility of AVNRT and a maximum of only single AV nodal reentrant echoes.

### Follow-up

Follow-up was obtained through outpatient clinic records as well as phone call or mail responses to a questionnaire specifically designed to assess for arrhythmia recurrence. For those with tachycardia recurrence, follow-up time was measured as the time in months from the ablation procedure to recurrence. For those free of recurrence, follow-up was measured as time from initial procedure to last contact. Six patients were lost to follow-up and were excluded from the analysis of LRR.

### Statistical analysis

We compared our acute procedural success, LRR, and complication rates to those reported in the major trials on both CTA and RFA for AVNRT. Data are presented as mean ± standard deviation.

## Results

### Clinical and demographic features

The clinical, demographic, and arrhythmic features of the participants are summarized in Table 1. In total, 11 patients (12.5 %) had structural heart disease including hypertensive heart disease, left ventricular systolic dysfunction, myxomatous mitral valve disease, mitral stenosis, aortic stenosis, right chambers dilatation associated with moderate pulmonary hypertension. Four patients had recurrence of AVNRT after prior RFA, and an additional patient had recurrence of AVNRT after a prior CTA undertaken at another institution.

**Table 1:** Baseline characteristics for CTA using TTT (*n* = 88)

Mean age	51.6 ± 17
Female	61 (69.3 %)
Male	27 (30.7 %)
Prior RFA for AVNRT	4 (4.5 %)
Prior CTA for AVNRT	1 (1.1 %)
Structural heart disease	11 (12.5 %)

### Procedural outcomes

The EP study and CTA outcomes are summarized in Table 2. Typical slow-fast AVNRT was observed in 79 patients (90.2 %), while 9 exhibited either the fast-slow or slow-slow varieties of AVNRT.

**Table 2:** Procedural outcomes for CTA using TTT (*n* = 88)

Acute procedural success	87 (98.9 %)
Cross over from CTA to RFA	1 (1.1 %)
Number of CTA applications	4.2 (3 to 21)
Residual dual AV nodal physiology	
None	44 (50 %)
Single echoes	43 (48.8 %)
Inducible AVNRT	1 (1.1 %)
Procedure time in minutes (mean ± SD)	155 ± 68
Complications	
Transient complete heart block	3 (3.4 %)
Transient second degree AV block	2 (2.3 %)
Permanent AV block	0 (0.0 %)

The number of CTM attempts before a successful TTT (≤20 s) was achieved ranged from 1 to 32. Forty-four patients (50.0 %) required ≤8 CTM attempts and 12 patients required only one. More than 10 CTM attempts were needed in only 9 patients (10.2 %). Successful CTM was achieved in ≤10 s in 30 patients and in 11 to 20 s in the remaining 57 patients.

A successful CTA using our strict TTT criteria of less than 20 s was achieved in 87 of the 88 patients (98.9 %). For the remaining 1 patient, the ablation modality was switched to RFA, which was successful at the same procedure session. In the 87 patients in whom CTA was successful, residual dual nodal physiology with single echoes was observed in 43 patients and dual pathway physiology was completely eliminated in the remaining 44 patients.

There were no early or late major procedural complications. No permanent AV block occurred, but transient AV block during CTM was noted in 8 patients (9.1 %) and promptly resolved within 30–180 s upon immediate re-warming. These sites were identified on the EAM system and were avoided during subsequent CTA applications. No further AV conduction disturbances were encountered.

Of the 87 patients in whom acute procedural success was achieved, 6 were lost to follow-up. Of the remaining 81 patients, 3 (3.7 %) experienced a recurrence of AVNRT during long-term follow-up. Time to recurrence ranged from 1 to 26 months (Table 3). The LRR is compared to the results of other CTA trials in Table 4.

**Table 3:** Long-term arrhythmic outcomes for CTA using TTT (*n* = 81)

Mean Follow-Up Duration (months)	19.7 ± 23.0
Range (months)	1–110
Late recurrence rate (documented AVNRT)	3.7 % (3/81)
Time to recurrence (months)	1, 1, 26

**Table 4:** Comparison of CTA using TTT to conventional CTA using 6-mm tip size

Author	Year	*N* (patients)	F/u (months)	Age (years)	Tip size (mm)	Acute success (%)	Late recurrence rate (%)	Permanent AV block (%)
Jensen-Urstad M^,^ et al. [8]	2006	75	9.2	53 ± 16	6	99	7	0
Khairy, et al. [9]	2007	185	24	49 ± 14	6	92	9	0
De Sisti, et al. [10]	2007	150	18 ± 10	39 ± 14	6	95	17	0
Chan, et al. [11]	2009	80	–	50 ± 12	6	97.5	9	0
Bastani, et al. [12]	2009	312	22 ± 10	22 ± 10	6	99	5.8	0
Deisenhofer, et al. [13]	2010	251	6	50 ± 15	6	97	9.4	0
CTA using TTT	2016	88	19.7 ± 23.0	51.6 ± 17	6	98.9	3.7	0

## Discussion

### Overview

In this study, we report our center’s acute and long-term procedural outcomes in 88 consecutive patients in whom we could use TTT as the guide for CTM success for AVNRT. CTA, as compared to RFA, offers patients enhanced safety from procedurally related permanent AV block. Nonetheless, CTA has not been the preferred method for ablation since its use has been associated with a significantly higher risk of LRR. In an effort to retain the safety advantage of CTA, we evaluated the hypothesis that a reduction in LRR could be achieved by more rigorously controlling the methodology of CTM. The changes to CTM that we employed included (1) performing CTM exclusively during sustained AVNRT and (2) requiring tachycardia termination in ≤20 s during CTM. Using this methodology for CTM, acute procedural success was excellent (98.9 %) and at a mean follow-up of over 19.7 months, the LRR of only 3.7 % was comparable to the best outcomes reported for patients undergoing RFA [13]. The results reported in this study suggest that strict control of the methods used for CTM is essential to achieve improved LRR. Furthermore, a possible explanation for the wide variation in LRR often reported for CTA of AVNRT may well derive from differences in the methodology employed for CTM (Table 4).

### Prior studies of CTA for AVNRT

Several prior studies of CTA for AVNRT have reported an unacceptably high LRR which ranged from 8 % to as high as 17 % [8–13]. Despite the use of apparently similar techniques and study populations, an explanation for this variation has been lacking. Some have attributed this variance to minor differences in technique, such as the use of “supplemental” CTA applications, or the use of larger sized catheter tips. It had been hoped that the introduction of the larger 6-mm tip, by providing larger and deeper lesions, would afford improved LRR. However, as summarized in Table 4, recent studies limited exclusively to the larger 6-mm tip have still reported LRR that were unacceptably high when compared to RFA [8–13]. For example, in the largest and most recent of these, the prospective randomized CYRANO trial, the clinical outcomes of 251 patients with AVNRT who underwent CTA with a 6-mm catheter tip were compared to those observed in 258 patients who were treated with standard RFA [13]. While acute procedural success was excellent in both groups, the LRR of 9.4 % observed for the CTA patients was significantly higher than the 4.4 % noted in the RFA group. As has been consistently reported, no permanent AV block was reported for patients randomized to CTA, while 3 patients (1 %) in the RFA group developed persistent AV block—one of whom required permanent pacing.

A full understanding of the CYRANO trial results, as well as those reported for other studies involving CTA, requires a careful examination of the precise methods utilized for the important variable of CTM. Often the techniques employed for CTM have been neither tightly controlled nor reported in detail. Important differences in the techniques of CTM could potentially explain why some studies have reported long-term outcomes for CTA equivalent to those seen for RFA, while the majority of trials have found RFA to be superior. Some important considerations in the performance of CTM are examined below.

### Specifics of CTM

The primary goal of CTM is to determine whether a specific catheter location is optimal for a permanent CTA lesion from the perspectives of both safety and efficacy. Of the many variables that have been shown to influence the results of CTM, two have recently been shown to have direct bearing on the likelihood of a long-term success. The first is the actual patient rhythm during which CTM is applied. For example, cooling during CTM can be applied during atrial pacing runs to assess slow pathway (SP) function, as is commonly chosen, or secondly, during induced sustained AVNRT. In the case of atrial pacing, the effect of CTM on SP conduction is assessed by repeated pacing runs directed at either inducing AVNRT or confirming impairment or elimination of SP function. When CTM is applied during induced sustained tachycardia, termination of AVNRT is the determinant of CTM success. It is likely that termination of AVNRT was a result of SP block as opposed to AV nodal block in that the integrity of both AV nodal and fast pathway conduction was confirmed immediately upon the resumption of sinus rhythm and, additionally, during subsequent atrial pacing runs applied shortly after tachycardia termination.

The second important variable in selecting an optimal ablation site is time: specifically, time to effect (TTE) defined as the duration of cooling to −30 ° C before the desired effect is achieved. In virtually all prior randomized trials comparing CTA and RFA, a TTE of up to 60 s was considered an acceptable duration [7, 13]. In this approach, either the lack of inducibility or the elimination/impairment of SP function, within the specified time frame of test cooling, is considered a favorable CTM result and indicates a site suitable for permanent CTA. The TTE at a given test site is a measure of catheter tip proximity to the targeted tissue. Given the smaller and more discrete lesion size associated with CTA, the use of a narrow window for TTE is likely to be indicative of greater proximity to the SP and a higher probability of achieving permanent interruption of SP function and long-term arrhythmia suppression.

In several recent studies, investigators have emphasized the importance of TTE during CTM as a determinant of LRR [12, 17]. In 30 pediatric patients with SVTs and right sided accessory pathways, Drago and colleagues were able to show a lower LRR of only 5.9 % when TTE was less 10 s, compared to greater than 20 % in those with a more prolonged TTE [17]. In a much larger study of 312 adults with slow-fast AVNRT, Bastani and colleagues strictly controlled CTM by limiting TTE to a maximum duration of 20 s. Acute procedural success was 99 %, and, importantly, the LRR improved to 5.8 % at an average follow-up of 6 years [12]. The observed decrease in LRR was in part attributed to the strict time criterion used to define a successful CTM response.

The ability to determine a precise TTE can be further enhanced by the performance of CTM during induced tachycardia. This approach allows for an unambiguous determination of TTE as indicated by the abrupt termination of tachycardia due to failure of SP conduction. In contrast, the performance of CTM during various atrial pacing maneuvers renders an accurate determination of TTE more difficult because the pacing maneuver itself consumes time. In addition, the ability to achieve consistent SP conduction is often variable from one pacing attempt to another, which further impedes the ability to determine a precise TTE. Lastly, the presence of multiple SPs is not infrequent and the prompt termination of tachycardia ensures that the pathway supporting the tachycardia is in fact the one being targeted for permanent CTA [20].

### Additional contributors to improved LRR

While the most likely explanations for the improved LRR observed in this study derive from the strict time-limited criterion used to guide CTM results and the performance of CTM during sustained AVNRT, other variables could have contributed as well. Three consecutively applied “consolidation” lesions were delivered. These applications were guided by EAM and located as close as possible to the initial successful site. In the CYRANO study, only one supplemental application was used [13]. The extent to which our additional consolidative CTA applications or the use of EAM contributed to our lower LRR is difficult to determine.

A remaining variable that could have influenced long-term outcome is that we routinely employed isoproterenol stimulation after an “apparently” successful initial CTA—even in patients who did not require its use for the induction of AVNRT in the baseline state. This additional step extended the duration of post CTA observation and, thereby, could have facilitated our ability to detect delayed recovery of SP function. In this study, we did not determine the extent to which this additional step led to further CTA applications.

### Study limitations

The primary limitation of our study is that it is a single-center, non-randomized trial. Additionally, our results are compared to those reported in previous trials—some recent, but also some more remote. Accordingly, it is possible that a patient selection bias may have in some way contributed to the favorable long-term outcome observed in our study population. While the sample study size of our study was modest, all of the 88 patients included in this study were consecutively enrolled and reflect all patients with AVNRT referred to our center for catheter ablation during the study interval.

Also, there are certain requirements inherent in the approach we employed in this study. First, it requires that patients exhibit, in a reproducible fashion, sustained AVNRT. This step will often necessitate the use of isoproterenol stimulation. In our experience, about 6–7 % of patients with documented AVNRT will fail to exhibit sustained tachycardia at the time of the procedure. In this situation, some other approach to CTM will be required. In such patients, CTM can usually be undertaken during atrial pacing maneuvers to assess impairment of SP function as described above [12, 13]. While it may be more difficult to determine a precise TTE, this approach can still be employed and used to achieve procedural success.

When compared to the CTA arm of the CYRANO trial, our procedure time was on average 14 min longer. This slightly longer procedure time can be largely accounted for by the 2 additional 4-min consolidating applications we employed and the use of isoproterenol infusion in all patients at the conclusion of the procedure. It is unlikely this small an extension of procedure time would have any significant clinical impact.

## Conclusions

Our study results suggest that CTA for AVNRT, contrary to the widely held perception, need not be associated with a higher likelihood of LRR. TTT represents a technical refinement when compared to conventional CTM. The performance of CTM during sustained AVNRT with the use of a strict TTT criterion of ≤20 s may have been the essential step needed to achieve this improvement. In fact, the LRR of only 3.7 % noted in our study is comparable to the 4.4 % rate noted in the recent CYRANO trial for RFA and is superior to the 9.4 % rate these investigators observed in their CTA group. Our findings, combined with those noted by others [12, 13], suggest that future trials of CTA for AVNRT should employ at least some of the strict CTM criteria used in our study.

These results will need to be confirmed, optimally in a controlled randomized trial, before a formal recommendation can be made. Admittedly, the risk of permanent AV block when RFA is used in this setting is low (0.4–2.0 %). However, given the large number of patients who are likely to undergo RFA for AVNRT in the future, many may be spared the risk of life-long permanent pacemaker dependence if CTA becomes the preferred initial approach to catheter ablation of AVNRT.

## Abbreviations

TTT: Time to tachycardia termination
TTE: Time to effect
AVNRT: Atrioventricular nodal reentry tachycardia
SP: Slow pathway
RFA: Radiofrequency ablation
EAM: Electro-anatomic mapping
CTA: Cryothermal ablation
CTM: Cryothermal mapping
LRR: Late recurrence rate
